# Effectiveness of benralizumab for allergic and eosinophilic predominant asthma following negative initial results with omalizumab

**DOI:** 10.1002/rcr2.388

**Published:** 2018-11-21

**Authors:** Daisuke Minami, Hiroe Kayatani, Ken Sato, Keiichi Fujiwara, Takuo Shibayama

**Affiliations:** ^1^ Department of Respiratory Medicine National Hospital Organization Okayama Medical Center Okayama City Okayama Japan

**Keywords:** Allergic, asthma, benralizumab, eosinophilic, omalizumab

## Abstract

A 64‐year‐old woman, who had presented with a 30‐year history of refractory asthma, and been treated with anti‐allergic drug therapy, inhaled corticosteroids, a long‐acting beta‐agonist, and a long‐acting muscarinic antagonist. She had been characterized as an allergic, eosinophilic asthmatic. Although omalizumab was tried initially, it was found to be insufficient. We began treatment with benralizumab. The asthma symptom control and sinusitis were improved immediately. Benralizumab was effective for overlapping patient population following negative initial results with omalizumab.

## Introduction

Benralizumab is an anti‐eosinophil monoclonal antibody against the interleukin‐5 (IL‐5) receptor. The drug significantly reduces annual exacerbation rates, and improves the forced expiratory volume in 1 second (FEV_1_) and asthma symptom control, while being well tolerated. The extent to which the exacerbation rate was reduced increased with the blood eosinophil threshold in a meta‐analysis of two studies 1, 2. However, little is known about the effectiveness of this therapy for combined allergic and eosinophilic predominant asthma. We present a patient with both allergic and eosinophilic predominant asthma whose asthma symptom control and sinusitis were improved by benralizumab following negative initial results with omalizumab.

## Case Report

A 64‐year‐old woman presented with a 30‐year history of refractory asthma. She had been treated with anti‐allergic drug therapy, inhaled corticosteroids (fluticasone propionate 1000 μg/day), a long‐acting beta‐agonist, and a long‐acting muscarinic antagonist. She had occasionally been treated with oral or systemic corticosteroids (oral prednisone 10 mg/day or methylprednisolone sodium succinate 80 mg/day, 3–5 days) for exertional dyspnoea. However, these treatments were all insufficient. She had been characterized as an allergic, eosinophilic asthmatic, with positive results for perennial inhalant allergen sensitivity, allergic rhinitis, and allergic diathesis (egg and sesame) with immunoglobulin E (IgE) and peripheral eosinophil levels of 618 IU/mL and 1006/μL, respectively, and omalizumab was tried initially. However, it was found to be insufficient owing to asthma exacerbation, and she required inpatient treatment 2 months later. In the 16th week following omalizumab, these treatments were not effective for asthma symptom control. If was felt that benralizumab was indicated for eosinophilic predominant asthma.

**Figure 1:**
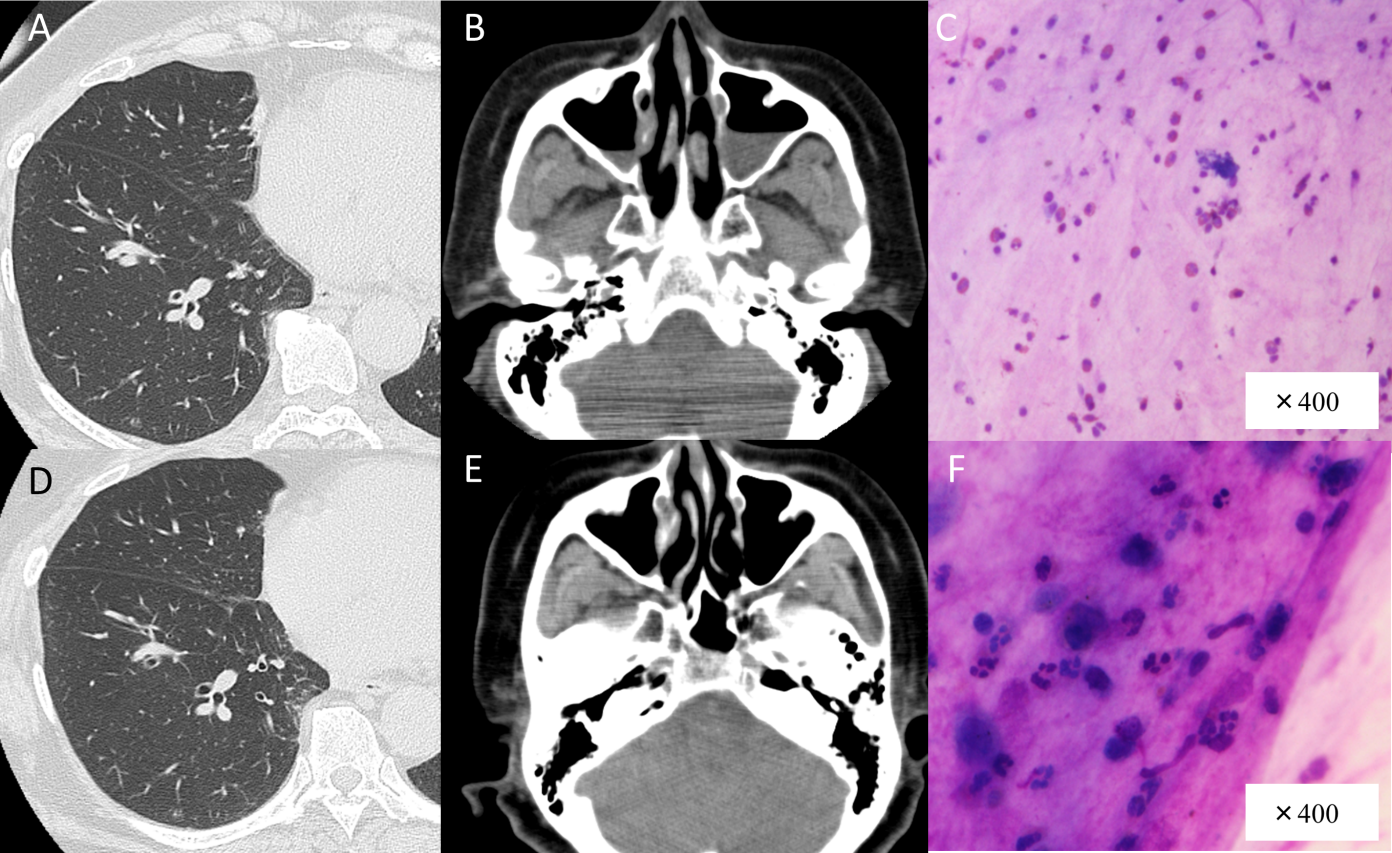
(A, B) Computed tomography shows diffuse bronchial wall thickening and sinusitis. (C) Sputum before benralizumab treatment revealed eosinophilia with Giemsa staining. (D, E) Computed tomography showed reduced mucus secretion in the bronchi and paranasal sinus 2 months later. (F) Eosinophils in sputum disappeared after benralizumab treatment. Sputum revealed neutrophilia on Giemsa staining.

On physical examination, chest auscultation revealed slight diffuse expiratory wheezing, but the remaining systemic examination did not reveal any significant abnormalities. Her peripheral arterial blood oxygen saturation was 95% on room air. Computed tomography (CT) showed diffuse bronchial wall thickening and paranasal sinusitis (Fig. 1A, B). She had sputum eosinophilia (Fig. 1C). The eosinophil count was more than 80% in sputum. Laboratory data showed an increase in the number of peripheral eosinophils and IgE level. The blood eosinophil count was 1924/μL and the IgE level was 4123 IU/mL. The patient’s FEV_1_ was 1640 mL (%FEV_1_, 66.3%) and her vital capacity (VC) was 2400 mL (%VC, 89.0%). The fractional exhaled nitric oxide (FeNO) was 13 ppb (Table 1).

**Table 1:** Time course of the benralizumab treatment.

	Before benralizumab	After benralizumab for 2 months	After benralizumab for 4 months
VC (mL)	2400	2490	2490
%VC (%)	89.0	91.7	93.0
FEV_1_ (mL)	1640	1760	1630
FEV_1_% (%)	66.3	73.3	70.1
%FEV_1_ (%)	80.2	85.6	80.7
FeNO (ppb)	13	26	15
Peripheral eosinophils (count/μL)	1924	0	0
Immunoglobulin E (IU/mL)	4123	1853	836
AQLQ score	5.53	6.15	6.43

Symptom improvement and fewer peripheral eosinophils were observed immediately after beginning benralizumab treatment.

AQLQ, Asthma Quality of Life Questionnaire; FeNO, fractional exhaled nitric oxide; FEV_1_, forced expiratory volume in 1 second; VC, vital capacity.

We began treatment with benralizumab. The eosinophil count decreased from 1924 to 0/μL after 2 weeks of treatment. The IgE level decreased from 4123 to 836 IU/mL after 4 months (Table 1). Moreover, CT showed reduced mucus secretion in the bronchus and paranasal sinus 2 months later (Fig. 1D, E). Eosinophils disappeared from the sputum immediately (Fig. 1F). However, the %VC, %FEV_1_, and FeNO did not change between baseline and 4 months following the initiation of benralizumab. The Asthma Quality of Life Questionnaire score improved from 5.53 at baseline to 6.43 4 months after treatment began (Table 1).

## Discussion

Only a minority of patients share equal features of both allergic and eosinophilic predominant asthma. In these patients with overlapping phenotypes, the treating physician usually chooses to start with either anti‐IgE or anti‐IL‐5 treatment. In patients with allergic asthma, the baseline blood eosinophil level (≥300/μL) predicts the response to omalizumab. Omalizumab reduces eosinophil numbers in peripheral blood and the airways of asthmatic patients based on clinical and observational studies and case reports 3. In patients with severe eosinophilic asthma not optimally controlled with omalizumab, when switched to mepolizumab, an anti‐eosinophil monoclonal antibody against the IL‐5 receptor, there was an improvement in asthma exacerbations, emergency department visits, and hospitalizations 4. In our case, benralizumab immediately improved the asthma symptom control and sinusitis after a negative initial response to omalizumab. Benralizumab strongly induces antibody‐dependent cell‐mediated cytotoxicity (ADCC) of eosinophils and basophils 5, and is effective for not only asthma symptom control but also sinusitis. In this overlapping patient population, there is no objective, evidence‐based answer to the question “Is anti‐IgE or anti‐IL‐5 the first choice?” A prospective large‐scale randomized study comparing these drugs might be of interest.

## Disclosure Statement

Appropriate written informed consent was obtained for publication of this case report and accompanying images.
